# Assessment of the unique nutrient contribution of white potatoes in the diet and the nutrient implications of replacing Refined and Whole Grains with Starchy Vegetables

**DOI:** 10.3389/fnut.2025.1692564

**Published:** 2025-11-27

**Authors:** Chesney Richter, Kristin Fulgoni, Victor L. Fulgoni, Bonnie Johnson, Michelle Kijek, Shelley Maniscalco, Tricia Psota

**Affiliations:** 1 Nutrition In Demand, Arlington, VA, United States; 2 Nutrition Impact, LLC, Battle Creek, MI, United States; 3 Potatoes USA, Denver, CO, United States

**Keywords:** dietary patterns, nutrient density, dietary guidelines, potatoes, starchy vegetables

## Abstract

**Background:**

Potatoes are a versatile and naturally nutrient-dense vegetable that contain many nutrients, including fiber, potassium, and vitamins C and B6. The 2025 Dietary Guidelines Advisory Committee (DGAC) considered changes to the Vegetable subgroups and potential interchangeability of Grains and Starchy Vegetables. This modeling analysis was conducted to quantify the nutrient contribution of white potatoes and Starchy Vegetables compared to Refined and Whole Grains.

**Methods:**

The objectives from the 2025–2030 DGAC protocol “What are the implications for nutrient intakes when modifying the quantities of the Grains group within the Healthy U.S.-Dietary Pattern” were modeled using the 2025 DGAC Staple Carbohydrate Foods Food Pattern Modeling (FPM) protocol and the 2020 DGAC FPM analyses. The modeling focused on women aged 19–30 years of age and men aged 51 + years of age to match the 2,000-calorie level widely used in dietary recommendations.

**Results:**

The recommended daily serving of white potatoes (0.58 cup equivalents) provides 11% of daily potassium and 6% of daily fiber, as well as vitamins B6 and C, copper, magnesium, thiamin, and niacin. White potatoes are currently underconsumed, while the current intake of Other Starchy Vegetables (e.g., corn, green peas, and plantains) nearly meets the recommended amount. Replacing Refined Grains with Starchy Vegetables increases fiber, potassium, vitamin C, and choline intake but decreases other nutrients such as iron, folate, and riboflavin. Replacing Whole Grains with Starchy Vegetables similarly increases potassium, vitamin C, and choline but decreases the intake of fiber, calcium, iron, magnesium, folate, and other vitamins.

**Conclusion:**

These results provide insight into the unique nutritional contribution of white potatoes and demonstrate that Starchy Vegetables and Grains are not interchangeable in dietary patterns based on their unique nutritional packages. Therefore, reducing the recommended intake of Starchy Vegetables may be counterproductive for improving diet quality.

## 1 Introduction

Potatoes are a versatile and naturally nutrient-dense vegetable that contain many nutrients, including complex carbohydrates, protein, fiber, iron, potassium, and vitamins C and B6. A medium potato provides 2 g of fiber (7% of the daily value) and 620 mg of potassium (15% of the daily value) (1)—both of which are defined as underconsumed nutrients of public health concern among the general U.S. population. In U.S. dietary guidance, potatoes are grouped with other starchy vegetables, such as corn and peas. The current *2020–2025 Dietary Guidelines for Americans* (DGA) recommends consuming five cup equivalents (cup eq.) per week of Starchy Vegetables, including white potatoes, as part of the Healthy U.S.-Style (HUSS) Dietary Pattern (2).

In addition to their nutrient density, potatoes are a favored vegetable among Americans. Starchy Vegetables account for over 25% of total vegetable intake (3, 4), and potatoes are the most commonly consumed vegetable (5). Observational research has also shown that potato consumption can be associated with improved diet quality (6, 7) and does not increase the risk of type 2 diabetes (8) or cardiovascular disease (9). Clinical studies have also demonstrated that potatoes can be included as part of a healthy diet with no negative cardiometabolic effects (10, 11). However, the Starchy Vegetables subgroup and total Vegetables remain underconsumed. According to the most recent population-level dietary intake data, 86% of Americans aged 2 years and older (12) consume less than the recommended amount of Starchy Vegetables.

As part of the development of the next iteration of DGA, the 2025 Dietary Guidelines Advisory Committee (DGAC) considered changes to the Vegetable subgroups and other “staple carbohydrate” foods. This included evaluating the interchangeability of Grain Foods and Starchy Vegetables based on their carbohydrate content (13). Ultimately, the 2025 DGAC Scientific Report and the proposed Eat Healthy Your Way dietary pattern recommended a one cup eq. reduction in the weekly servings of Starchy Vegetables to allow for an increase in servings of Beans, Peas, and Lentils (12). Decreasing the recommended amount of a favored, nutrient-dense, and underconsumed vegetable subgroup in a healthy dietary pattern could have negative implications for nutrient intakes across the U.S. population.

This modeling analysis was conducted to quantify the nutrient contribution of White Potatoes and Starchy Vegetables compared to other carbohydrate-rich foods (i.e., Refined and Whole Grains). The primary aim was to better understand the nutrient implications of substituting or removing Starchy Vegetables in dietary patterns. An additional objective was to examine the impact of replacing Refined Grains and/or Whole Grains with Starchy Vegetables, as both are commonly consumed carbohydrate-rich foods.

## 2 Materials and methods

The following objectives from the 2025–2030 DGAC protocol “What are the implications for nutrient intakes when modifying the quantities of the Grains group within the Healthy U.S.-Dietary Pattern” were included within the study (14). The modeling focused on women aged 19–30 years of age and men aged 51 + years of age to match the 2,000-calorie level widely used in dietary recommendations.

- Objective 1: Identify the nutritional composition and contribution of Grains and other “staple carbohydrate foods” (i.e., Whole Grains, Refined Grains, White Potatoes, Other Starchy Vegetables) in current dietary intakes, relative to the 2020 HUSS Dietary Pattern goals.
- Objective 2: Compare the nutrient profiles of the Grains food group to the nutrient profiles of the Starchy Vegetable subgroups (i.e., White Potatoes and Other Starchy Vegetables).
- Objective 3: Evaluate implications on meeting nutritional goals when the proportions of Grains subgroups (i.e., Refined Grains and Whole Grains) in the 2020 HUSS Dietary Pattern are individually reduced by 1/2 or 1 oz. equivalent (oz eq.) increments and when the proportions of other staple carbohydrate foods (i.e., White Potatoes and Other Starchy Vegetables) are increased by 1/4 or 1/2 cup eq. increments. The process will model various subgroup proportions to represent potential levels of consumption. The nutritional composition and contributions of the modifications of staple carbohydrate foods will be examined.
- Objective 4: Evaluate implications on meeting nutritional goals when both Grains subgroups (i.e., Whole Grains and Refined Grains) in the 2020 HUSS Dietary Pattern are reduced by 1 or 2 oz. eq. increments and when the proportions of other staple carbohydrate foods (i.e., White Potatoes and Other Starchy Vegetables) are increased by 1/2 to 1 cup eq. increments. The process will model various subgroup proportions to represent potential levels of consumption. The nutritional composition and contributions of the modifications to the Grains and Vegetables food groups will be examined.

### 2.1 Modeling objective 1

To complete the objectives, the current study protocols were modeled after the 2025 DGAC Staple Carbohydrate Foods Food Pattern Modeling (FPM) (14) protocol and 2020 DGAC FPM analyses (3). The nutrient content of Whole Grains, Refined Grains, White Potatoes, and Other Starchy Vegetable subgroups was calculated by identifying Standard Reference Legacy codes that matched the representative foods in the respective sections of Table 4.1 from the FPM report. The number of representative foods included for each subgroup was as follows: Whole Grains, 15 representative foods; Refined Grains, 16 representative foods; White Potatoes, 5 representative foods; and Other Starchy Vegetables, 8 representative foods. USDA’s FoodData Central was used as the source of nutrient values, and the Food Pattern Equivalents Database was utilized to convert cup eq. to grams (1, 15). The nutrient values for the individual representative foods were multiplied by the percentage of the food subgroup they represented. The nutrient content of the subgroups was determined by summing the above calculations.

For White Potatoes and Other Starchy Vegetables, the percentage of the subgroup represented by individual representative foods had to be determined. This was performed by dividing the percent subgroup (from Table 4.1 of the FPM report) of individual representative foods by the sum of all other representative foods within the White Potatoes/Other Starchy Vegetables subgroup.

For example, french fries as part of the White Potatoes subgroup:

- Table 4.1 2020 DGAC FPM document—Item cluster: french fries. Representative Food: white potato, french fries, from frozen, oven baked
- Nutrient values were collected from FoodData Central for Potato, french fries, all types, salt not added in processing, frozen, and oven-heated
- Food Pattern Equivalents Database—Potato, french fries, from frozen conversion is 1 cup eq. = 154 grams
- French fries represented 13.5% of the Starchy Vegetable subgroup (Table 4.1 of the FPM report). Home fries/hashbrowns, potato chips/puffs/sticks, baked potatoes, and boiled potatoes represented 8.5, 18.8, 15.6, and 26.5% of the Starchy Vegetable subgroup, respectively.
- The percentage of the White Potato subgroup represented by french fries = 13.5/(8.5 + 18.8 + 15.6 + 26.5 + 13.5) = 16.3%
- To determine the number of calories in the White Potato subgroup provided by french fries, the number of calories in 1 cup eq. of french fries was multiplied by the percentage of the White Potato subgroup represented by french fries. This calculation was repeated for home fries/hashbrowns, potato chips/puffs/sticks, baked potatoes, and boiled potatoes, and the values were summed to get the number of calories in 1 cup eq. of the new White Potatoes subgroup.

#### 2.1.1 Contribution to dietary pattern

To determine the contribution of these subgroups in the HUSS Dietary Pattern, recommended amounts of food from each food group at the 2,000-calorie level and consumption-weighted nutrition profile for the HUSS Dietary Pattern were obtained from Table 3.1 and Table 5.5 in the FPM document, respectively. The recommended intake for White Potatoes and Other Starchy Vegetables requires computation. The percentage of the food group represented by the foods of interest (identified from Table 4.1 of the FPM report) was multiplied by the total Vegetable recommendation. For example: french fries, home fries, potato chips, baked potatoes, and boiled potatoes represented a total of 23.1% of the Vegetable food group, and the total Vegetable recommendation for the 2,000-calorie level is 2.5 cups. Therefore, the calculated recommended intake of White Potatoes is 0.58 cup eq. The recommended servings of Whole Grains, Refined Grains, White Potatoes, and Other Starchy Vegetables were multiplied by the calculated nutrient content of the respective subgroups. This value was divided by the consumption-weighted nutrition profile of the dietary pattern to determine the contribution of the subgroups in the healthy dietary pattern.

#### 2.1.2 Contribution to current intake

Data from Tables 2.6, 2.11, 2.13, and 2.14 of the Food Group and Nutrient Distribution: All Life Stages 2020 DGAC Supplementary Data Analysis (16) were utilized to determine the nutrient contribution of the Whole Grains, Refined Grains, White Potatoes, and Other Starchy Vegetables subgroup in the current intake. The nutrient values of the recommended subgroup servings were replaced with the current intake of the subgroups and then divided by the consumption-weighted nutrition profile of the dietary pattern to determine the contribution of the subgroup in current dietary intake.

### 2.2 Modeling objective 2

Objective 2 was completed by calculating the nutrient content difference between individual subgroups. Two ounces of total Grains (calculated as the sum of 1 oz. of Whole and Refined Grains) was compared to 1 cup eq. of White Potatoes and Starchy Vegetables, as recommended by the 2025 DGAC grains FPM protocol (14).

### 2.3 Modeling objective 3

Nutritional goals were sourced from Table 2.1 in the FPM report (3), and the current pattern (baseline) nutrient intake was sourced from Table 5.5. The nutrient values of the Refined/Whole Grain subgroups calculated in objective 1 were utilized. Replacements were simulated by subtracting the nutrient content of 2 oz. of Refined/Whole Grains from the baseline and adding the nutrients provided by 1 cup eq. of Starchy Vegetables (calculated following the protocol from objective 1). This was then divided by nutritional goals for the 2,000-calorie level and was repeated for a 10- and 21-oz reduction of Refined/Whole Grains and a 5- and 10.5-cup eq. increase in Starchy Vegetables, respectively. Scenario selection reasoning was as follows: replacement of 2 oz. of Refined/Whole Grains with 1 cup eq. of Starchy Vegetables simulated increasing Starchy Vegetables by 1 cup eq. per week; replacement of 10 oz. of Refined/Whole Grains with 5 cup eq. of Starchy Vegetables simulated doubling the current recommendation; and replacement of 21 oz. of Refined/Whole Grains with 10.5 cup eq. of Starchy Vegetables simulated total replacement. Nutrient values less than 75 and 90% of intake recommendations were highlighted because, in many cases, USDA flags these levels to run additional modeling.

## 3 Results

### 3.1 Modeling objective 1: nutritional composition and contribution of food subgroups (Whole Grains, Refined Grains, White Potatoes, and Other Starchy Vegetables)

The recommended daily servings of White Potatoes contribute 11% of the Dietary Reference Intake (DRI) for potassium, 10% for vitamin B6, and 9% for copper at only 5% of daily calories and 3% of sodium. White Potatoes also provide 5% or more of daily fiber, magnesium, vitamin C, thiamin, and niacin (Table 1). While Whole Grains provide substantially more fiber (23%), the amount of fiber provided by Refined Grains is similar to that of White Potatoes. The amount of potassium provided by White Potatoes is 38% higher than that provided by Whole Grains and over three times the amount provided by Refined Grains.

**Table 1:** Nutrient contribution of the recommended daily servings of Whole Grains, Refined Grains, White Potatoes, and Other Starchy Vegetables in the Healthy U.S.-Style (HUSS) Dietary Pattern.^a^

	Whole Grains (oz-eq)		Refined Grains (oz-eq)		White Potatoes (cup-eq)		Other Starchy Vegetables (cup-eq)	
Recommended daily servings	3		3		0.58^b^		0.12^b^	
	Amount	%	Amount	%	Amount	%	Amount	%
Macronutrients								
Calories (kcal)	273.87	**14%**	259.41	*13%*	107.25	5%	18.69	1%
Protein (g)	9.87	**11%**	7.14	8%	2.06	2%	0.69	1%
Total fat (g)	4.77	**7%**	3.84	5%	1.79	3%	0.18	0%
Carbohydrate (g)	50.13	**19%**	48.66	*19%*	21.28	8%	4.07	2%
Fiber (g)	6.81	**23%†**	2.13	7%	1.91	6%	0.60	2%
Cholesterol (mg)	1.32	1%	1.20	1%	0.00	0%	0.00	0%
Saturated fat (g)	0.9	5%	1.02	6%	0.25	1%	0.02	0%
Monounsaturated fat (g)	1.2	5%	1.29	5%	1.07	4%	0.04	0%
Polyunsaturated fat (g)	2.1	**10%**	1.08	5%	0.31	1%	0.07	0%
Minerals								
Calcium (mg)	105.3	8%	70.77	6%	8.68	1%	2.22	0%
Iron (mg)	4.71	**34%†**	2.88	**21%**	0.55	4%	0.15	1%
Magnesium (mg)	87.18	**24%†**	23.31	7%	20.26	6%	5.96	2%
Phosphorus (mg)	250.83	**15%**	116.37	7%	50.94	3%	15.55	1%
Potassium (mg)	273.84	8%	98.82	3%	372.42	**11%**	50.50	1%
Sodium (mg)	224.55	**14%**	314.22	*19%*	56.03	3%	0.64	0%
Zinc (mg)	3.09	**24%†**	0.66	5%	0.26	2%	0.13	1%
Copper (mg)	0.27	**19%**	0.12	9%	0.12	9%	0.02	1%
Selenium (mcg)	20.49	**18%**	18.93	*17%*	0.80	1%	0.13	0%
Vitamins								
Vitamin A (mcg RAE)	87.81	**10%**	24.60	3%	0.08	0%	3.64	0%
Vitamin E (mg AT)	0.99	**10%**	0.36	4%	0.21	2%	0.02	0%
Vitamin D (IU)	12.39	4%	6.42	2%	0.00	0%	0.00	0%
Vitamin C (mg)	1.92	1%	1.56	1%	6.95	5%	1.59	1%
Thiamin (mg)	0.36	**20%†**	0.48	**27%†**	0.15	8%	0.02	1%
Riboflavin (mg)	0.12	6%	0.24	*12%*	0.02	1%	0.02	1%
Niacin (mg)	4.62	**20%†**	3.66	*16%*	1.55	7%	0.30	1%
Vitamin B6 (mg)	0.3	**14%**	0.12	5%	0.22	*10%*	0.03	1%
Vitamin B-12 (mcg)	0.6	**10%**	0.15	2%	0.00	0%	0.00	0%
Choline (mg)	22.35	6%	10.17	3%	11.59	3%	5.20	1%
Vitamin K (mcg)	4.26	3%	1.80	1%	3.18	2%	1.38	1%
Folate (mcg DFE)	131.37	**26%†**	131.49	**26%**	12.47	2%	6.36	1%

a Data presented are for women aged 19–30 years and men aged 51 + years at the 2,000-calorie level.

b Recommendations for White Potatoes and Other Starchy Vegetables were calculated by summing the percentage of the food group represented by foods of interest and multiplying that by the total Vegetable recommendation for the age/gender group. For example: french fries, home fries, potato chips, baked potatoes, and boiled potatoes represented a total of 23.1% of the Vegetable food group, and the total Vegetable recommendation for the 2,000-calorie level is 2.5 cups. Therefore, the calculated recommended intake of White Potatoes is 0.58 cup eq. Bold indicates ≥10% of expected daily nutrients in the HUSS Dietary Pattern provided by the recommended subgroup serving. Bold† indicates ≥20% of expected daily nutrients in the HUSS Dietary Pattern provided by the recommended subgroup serving.

The current intakes of each food subgroup (e.g., Whole Grains, Refined Grains, White Potatoes, and Other Starchy Vegetables) and their nutrient contribution are shown in Table 2. Both women 19–30 years and men 51 + years consume over 1.5 times the recommended daily amount of Refined Grains. Whole Grain consumption is only 27 and 40% of the recommended amount for women and men, respectively. White Potatoes are also underconsumed, with women aged 19–30 years and men aged 51 + years consuming only 52 and 69% of calculated White Potato recommendations, respectively. The current intake of White Potatoes provides 6% of daily potassium for women and 8% of daily potassium for men, which is less than the 11% that would be provided by the recommended amount. Conversely, the current intake of Other Starchy Vegetables (e.g., corn, green peas, plantains) (0.1 cup eq.) nearly meets the recommended daily amount of 0.12 cup eq.

**Table 2:** Percent of nutrients contributed by the current intake of Whole Grains, Refined Grains, White Potatoes, and Other Starchy Vegetables in the Healthy U.S.-Style (HUSS) dietary pattern.^a^

	Whole Grains		Refined Grains		White Potatoes		Other Starchy Vegetables	
	Women 19–30 y	Men 51 + y	Women 19–30 y	Men 51 + y	Women 19–30 y	Men 51 + y	Women 19–30 y	Men 51 + y
Recommended daily intake	3 oz. eq		3 oz. eq		0.58 cup eq		0.12 cup eq	
Current daily intake	0.8 oz. eq	1.2 oz. eq	5.3 oz. eq	5.65 oz. eq	0.3 cup eq	0.4 cup eq	0.1 cup eq	0.1 cup eq
Macronutrients								
Calories (kcal)	4%	6%	**21%†**	**22%†**	3%	4%	1%	1%
Protein (g)	3%	5%	**13%**	**14%**	1%	2%	1%	1%
Total fat (g)	2%	3%	9%	*10%*	1%	2%	0%	0%
Carbohydrate (g)	6%	9%	**29%†**	**30%†**	4%	6%	1%	1%
Fiber (g)	7%	**11%**	**12%**	**13%**	3%	4%	2%	2%
Cholesterol (mg)	0%	0%	1%	1%	0%	0%	0%	0%
Saturated fat (g)	1%	2%	**10%**	**10%**	1%	1%	0%	0%
Monounsaturated fat (g)	1%	2%	9%	9%	2%	3%	0%	0%
Polyunsaturated fat (g)	3%	4%	8%	9%	1%	1%	0%	0%
Minerals								
Calcium (mg)	2%	3%	9%	**10%**	0%	0%	0%	0%
Iron (mg)	**12%**	**17%**	**31%†**	**33%†**	2%	3%	1%	1%
Magnesium (mg)	8%	**11%**	**11%**	**12%**	3%	4%	1%	1%
Phosphorus (mg)	5%	7%	**12%**	**12%**	2%	2%	1%	1%
Potassium (mg)	2%	3%	5%	5%	6%	8%	1%	1%
Sodium (mg)	4%	6%	**29%†**	**31%†**	2%	2%	0%	0%
Zinc (mg)	8%	**11%**	9%	9%	1%	1%	1%	1%
Copper (mg)	6%	9%	**14%**	**15%**	5%	6%	1%	1%
Selenium (mcg)	6%	8%	**26%†**	**27%†**	0%	0%	0%	0%
Vitamins								
Vitamin A (mcg RAE)	3%	4%	5%	5%	0%	0%	0%	0%
Vitamin E (mg AT)	3%	4%	6%	7%	1%	1%	0%	0%
Vitamin D (IU)	1%	2%	4%	4%	0%	0%	0%	0%
Vitamin C (mg)	0%	1%	2%	2%	3%	4%	1%	1%
Thiamin (mg)	6%	9%	**39%†**	**41%†**	5%	6%	1%	1%
Riboflavin (mg)	2%	2%	**19%**	**20%†**	1%	1%	1%	1%
Niacin (mg)	6%	9%	**25%†**	**26%†**	4%	5%	1%	1%
Vitamin B6 (mg)	4%	6%	9%	**10%**	5%	7%	1%	1%
Vitamin B-12 (mcg)	3%	4%	4%	4%	0%	0%	0%	0%
Choline (mg)	2%	3%	5%	5%	2%	2%	1%	1%
Vitamin K (mcg)	1%	1%	2%	2%	1%	2%	1%	1%
Folate (mcg DFE)	8%	**12%**	**38%†**	**39%†**	1%	2%	1%	1%

a Data presented are for women 19–30 years and men 51 + years at the 2,000-calorie level. Bold indicates ≥10% of expected daily nutrients in the HUSS Dietary Pattern provided by current subgroup intake. Bold† indicates ≥20% of expected daily nutrients in the HUSS Dietary Pattern provided by current subgroup intake.

### 3.2 Modeling objective 2: comparison of nutrient profiles (Grains, White Potatoes, and Other Starchy Vegetables)

**Figure 1:**
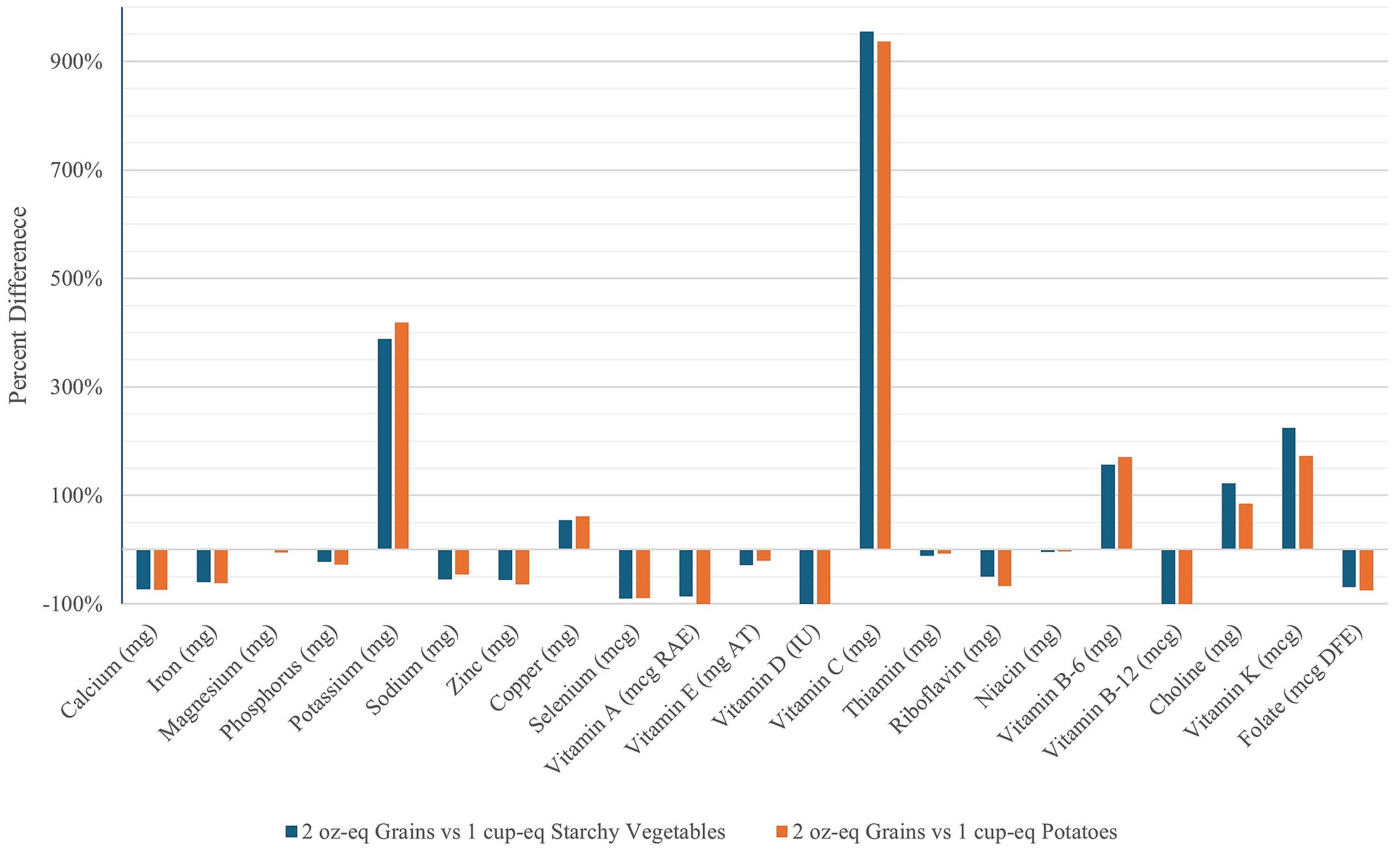
Percent difference of vitamin and mineral content of Starchy Vegetables and White Potatoes (1 cup-eq.) compared to Grains (2 oz-eq.).

A one-cup-eq. serving of White Potatoes or Starchy Vegetables provides over 350% more potassium and over 900% more vitamin C than a two-oz.-eq. portion of Grains, but less calcium, iron, zinc, selenium, riboflavin, folate, and vitamins A, D, and B-12 (Figure 1). Absolute differences in amounts of macronutrients and micronutrients are provided in Supplementary Table 1.

### 3.3 Modeling objective 3: nutritional implications of modifications to Refined Grains or Whole Grains and Starchy Vegetables quantities

The current reference pattern (i.e., the HUSS Dietary Pattern at the 2,000-calorie level) provided at least 90% of recommended intake or exceeded nutrient recommendations for the majority of nutrients except sodium, choline, and vitamins D and E in both women aged 19–30 years (Table 3) and men aged 51 + years (Table 4). In women, the reference pattern provided only 78% of the iron recommendations, and in men, it provided only 85% of the nutrient recommendations for magnesium.

**Table 3:** Nutrient implications of replacing Refined Grains with Starchy Vegetables in women 19–30 years (2,000 kcal)^a^.

	Current pattern (reference)		Refined Grains: −2 oz-eq Starchy Vegetables: +1 cup-eq		Refined Grains: −10 oz-eq Starchy Vegetables: +5 cup-eq		Refined Grains: −21 oz-eq Starchy Vegetables: +10.5 cup-eq	
Refined Grains (oz-eq/wk)	21		19		11		0	
Whole Grains (oz-eq/wk)	21		21		21		21	
Starchy Vegetables (cup-eq/wk)	5		6		10		15.5	
	Intake	% RDA/AI	Intake	% RDA/AI	Intake	% RDA/AI	Intake	% RDA/AI
Macronutrients								
Calories (kcal)	2001	100%	2008.6	100%	2038.8	102%	2080.4	104%
Protein (g)	92	200%	91.2	198%	88.0	191%	83.5	182%
Total fat (g)	71		71.3		72.3		73.7	
Carbohydrate (g)	259	199%	262.9	202%	278.6	214%	300.2	231%
Fiber (g)	30	107%	32.2	115%	40.9	146%	52.8	189%
Cholesterol (mg)	214	**71%†**	213.2	**71%†**	210.0	**70%†**	205.6	**69%†**
Saturated fat (g)	18	–	17.7	–	16.6	–	15.0	–
% of daily calories		8.1%		7.9%		7.3%		6.5%
Monounsaturated fat (g)	25	–	25.7	–	28.7	–	32.7	–
Polyunsaturated fat (g)	22	–	21.8	–	21.2	–	20.2	–
Minerals								
Calcium (mg)	1,278	128%	1246.5	125%	1120.3	112%	946.7	95%
Iron (mg)	14	**78%**	13.1	**73%†**	9.4	**52%†**	4.3	**24%†**
Magnesium (mg)	358	115%	380.0	123%	468.1	151%	589.2	190%
Phosphorus (mg)	1,654	236%	1671.7	239%	1742.5	249%	1839.7	263%
Potassium (mg)	3,390	130%	3930.0	151%	6090.0	234%	9060.0	348%
Sodium (mg)	1,658	**72%†**	1529.7	**67%†**	1016.6	**44%†**	311.0	**14%†**
Zinc (mg)	13	163%	13.1	164%	13.6	169%	14.2	177%
Copper (mg)	1.4	156%	1.5	169%	2.0	222%	2.7	296%
Selenium (mcg)	113	205%	101.7	185%	56.6	103%	−5.4	**0%†**
Vitamins								
Vitamin A (mcg RAE)	898	128%	887.0	127%	842.8	120%	782.0	112%
Vitamin E (mg AT)	10	**67%†**	10.1	**67%†**	10.4	**69%†**	10.8	**72%†**
Vitamin D (IU)	300	**50%†**	295.7	**49%†**	278.6	**46%†**	255.1	**43%†**
Vitamin C (mg)	129	172%	140.2	187%	185.0	247%	246.6	329%
Thiamin (mg)	1.8	164%	1.7	157%	1.5	132%	1.1	97%
Riboflavin (mg)	2	182%	1.9	173%	1.5	136%	1.0	**86%**
Niacin (mg)	23	164%	23.2	166%	24.1	172%	25.2	180%
Vitamin B6 (mg)	2.2	169%	2.5	191%	3.6	277%	5.1	395%
Vitamin B-12 (mcg)	6.2	258%	6.1	254%	5.7	238%	5.2	215%
Choline (mg)	355	**84%**	372.3	**88%**	441.5	104%	536.7	126%
Vitamin K (mcg)	140	156%	145.3	161%	166.7	185%	196.1	218%
Folate (mcg DFE)	513	128%	452.4	113%	210.1	**53%†**	−123.2	**0%†**

a Bold indicates between 75% and 90% of the recommended nutrient intake provided by the total diet. Bold† indicates below 75% of the recommended nutrient intake provided by the total diet. The percent of daily calories from saturated fat is provided in place of the RDA/AI, as the *Dietary Guidelines for Americans, 2020–2025* recommends limiting saturated fat intake to <10% of daily calories.

**Table 4:** Nutrient implications of replacing Refined Grains with Starchy Vegetables in men aged 51 + years (2,000 kcal)^a^.

	Current pattern (reference)		Refined Grains: −2 oz-eq Starchy Vegetables: +1 cup-eq		Refined Grains: −10 oz-eq Starchy Vegetables: +5 cup-eq		Refined Grains: −21 oz-eq Starchy Vegetables: +10.5 cup-eq	
Refined Grains (oz-eq/wk)	21		19		11		0	
Whole Grains (oz-eq/wk)	21		21		21		21	
Starchy Vegetables (cup-eq/wk)	5		6		10		15.5	
	Intake	% RDA/AI	Intake	% RDA/AI	Intake	% RDA/AI	Intake	% RDA/AI
Macronutrients								
Calories (kcal)	2001	100%	2008.6	100%	2038.8	102%	2080.4	104%
Protein (g)	92	164%	91.2	163%	88.0	157%	83.5	149%
Total fat (g)	71		71.3		72.3		73.7	
Carbohydrate (g)	259	199%	262.9	202%	278.6	214%	300.2	231%
Fiber (g)	30	107%	32.2	115%	40.9	146%	52.8	189%
Cholesterol (mg)	214	**71%†**	213.2	**71%†**	210.0	**70%†**	205.6	**69%†**
Saturated fat (g)	18		17.7	–	16.6	–	15.0	–
% of daily calories		8.1%		7.9%		7.3%		6.5%
Monounsaturated fat (g)	25	–	25.7	–	28.7	–	32.7	–
Polyunsaturated fat (g)	22	–	21.8	–	21.2	–	20.2	–
Minerals								
Calcium (mg)	1,278	128%	1246.5	125%	1120.3	112%	946.7	95%
Iron (mg)	14	175%	13.1	164%	9.4	118%	4.3	**54%†**
Magnesium (mg)	358	**85%**	380.0	**90%**	468.1	111%	589.2	140%
Phosphorus (mg)	1,654	236%	1671.7	239%	1742.5	249%	1839.7	263%
Potassium (mg)	3,390	100%	3930.0	116%	6090.0	179%	9060.0	266%
Sodium (mg)	1,658	**72%†**	1529.7	**67%†**	1016.6	**44%†**	311.0	**14%†**
Zinc (mg)	13	118%	13.1	119%	13.6	123%	14.2	129%
Copper (mg)	1.4	156%	1.5	169%	2.0	222%	2.7	296%
Selenium (mcg)	113	205%	101.7	185%	56.6	103%	−5.4	**0%†**
Vitamins								
Vitamin A (mcg RAE)	898	100%	887.0	99%	842.8	94%	782.0	**87%**
Vitamin E (mg AT)	10	**67%†**	10.1	**67%†**	10.4	**69%†**	10.8	**72%†**
Vitamin D (IU)	300	**50%†**	295.7	**49%†**	278.6	**46%†**	255.1	**43%†**
Vitamin C (mg)	129	143%	140.2	156%	185.0	206%	246.6	274%
Thiamin (mg)	1.8	150%	1.7	144%	1.5	121%	1.1	**89%**
Riboflavin (mg)	2	154%	1.9	146%	1.5	115%	1.0	**73%†**
Niacin (mg)	23	144%	23.2	145%	24.1	150%	25.2	158%
Vitamin B6 (mg)	2.2	129%	2.5	146%	3.6	212%	5.1	302%
Vitamin B-12 (mcg)	6.2	258%	6.1	254%	5.7	238%	5.2	215%
Choline (mg)	355	**65%†**	372.3	**68%†**	441.5	**80%**	536.7	98%
Vitamin K (mcg)	140	117%	145.3	121%	166.7	139%	196.1	163%
Folate (mcg DFE)	513	128%	452.4	113%	210.1	**53%†**	−123.2	**0%†**

a Bold indicates between 75% and 90% of the recommended nutrient intake provided by the total diet. Bold† indicates below 75% of the recommended nutrient intake provided by the total diet. The percent of daily calories from saturated fat is provided in place of the RDA/AI, as the *Dietary Guidelines for Americans, 2020–2025* recommends limiting saturated fat intake to <10% of daily calories.

Overall, for both women and men, the replacement of Refined Grains with Starchy Vegetables increased the percentage of nutrient recommendations achieved for choline, magnesium, and vitamin E and decreased the percentage achieved for iron, sodium, selenium, folate, riboflavin, and vitamin D (Tables 3, 4). Potassium, vitamins B6 and C, and fiber also increased at all levels of replacement, but these nutrients already met or exceeded the recommended amount in the current HUSS reference pattern.

The replacement of Whole Grains with Starchy Vegetables increased the percentage of choline recommendations achieved and decreased the percentage for fiber, calcium, iron, magnesium, sodium, zinc, selenium, folate, and vitamins A, B-12, E, and D in both women aged 19–30 years (Table 5) and men aged 51 + years (Table 6). Potassium, copper, and vitamins B6 and C also increased at all levels of replacement in both women and men, but their intakes already met or exceeded the recommended amount in the reference dietary pattern.

**Table 5:** Nutrient implications of replacing Whole Grains with Starchy Vegetables in women aged 19–30 years (2,000 kcal)^a^.

	Current pattern (reference)		Whole Grains: −2 oz-eq Starchy Vegetables: +1 cup-eq		Whole Grains: −10 oz-eq Starchy Vegetables: +5 cup-eq		Whole Grains: −21 oz-eq Starchy Vegetables: +10.5 cup-eq	
Refined Grains (oz-eq/wk)	21		21		21		21	
Whole Grains (oz-eq/wk)	21		19		11		0	
Starchy Vegetables (cup-eq/wk)	5		6		10		15.5	
	Intake	% RDA/AI	Intake	% RDA/AI	Intake	% RDA/AI	Intake	% RDA/AI
Macronutrients								
Calories (kcal)	2001	100%	1998.9	100%	1990.6	100%	1979.2	99%
Protein (g)	92	200%	89.4	194%	78.9	171%	64.4	140%
Total fat (g)	71		70.6		69.2		67.2	
Carbohydrate (g)	259	199%	261.9	201%	273.7	211%	289.9	223%
Fiber (g)	30	107%	29.1	104%	25.3	**90%**	20.0	**72%†**
Cholesterol (mg)	214	**71%†**	213.1	**71%†**	209.6	**70%†**	204.8	**68%†**
Saturated fat (g)	18	–	17.8	–	17.0	–	15.8	–
% of daily calories		8.1%		8.0%		7.7%		7.2%
Monounsaturated fat (g)	25	–	25.8	–	29.0	–	33.3	–
Polyunsaturated fat (g)	22	–	21.2	–	17.8	–	13.1	–
Minerals								
Calcium (mg)	1,278	128%	1223.4	122%	1005.2	101%	705.0	**71%†**
Iron (mg)	14	**78%**	11.9	**66%†**	3.3	**18%†**	−8.5	**0%†**
Magnesium (mg)	358	115%	337.4	109%	255.2	**82%**	142.1	**46%†**
Phosphorus (mg)	1,654	236%	1582.1	226%	1294.3	185%	898.5	128%
Potassium (mg)	3,390	130%	3813.3	147%	5506.6	212%	7834.9	301%
Sodium (mg)	1,658	**72%†**	1589.5	**69%†**	1315.5	**57%†**	938.6	**41%†**
Zinc (mg)	13	163%	11.5	144%	5.5	**68%†**	−2.9	**0%†**
Copper (mg)	1.4	156%	1.4	158%	1.5	167%	1.6	179%
Selenium (mcg)	113	205%	100.7	183%	51.4	93%	−16.4	**0%†**
Vitamins								
Vitamin A (mcg RAE)	898	128%	844.8	121%	632.1	**90%**	339.5	**49%†**
Vitamin E (mg AT)	10	**67%†**	9.7	**64%†**	8.3	**55%†**	6.4	**43%†**
Vitamin D (IU)	300	**50%†**	291.7	**49%†**	258.7	**43%†**	213.3	**36%†**
Vitamin C (mg)	129	172%	140.0	187%	183.8	245%	244.1	325%
Thiamin (mg)	1.8	164%	1.8	165%	1.9	168%	1.9	173%
Riboflavin (mg)	2	182%	2.0	180%	1.9	173%	1.8	163%
Niacin (mg)	23	164%	22.6	161%	20.9	149%	18.5	132%
Vitamin B6 (mg)	2.2	169%	2.4	182%	3.0	231%	3.9	298%
Vitamin B-12 (mcg)	6.2	258%	5.8	242%	4.2	175%	2.0	**83%**
Choline (mg)	355	**84%**	364.2	**86%**	400.9	94%	451.4	106%
Vitamin K (mcg)	140	156%	143.7	160%	158.5	176%	178.9	199%
Folate (mcg DFE)	513	128%	452.5	113%	210.5	**53%†**	−122.4	**0%†**

a Bold indicates between 75% and 90% of the recommended nutrient intake provided by the total diet. Bold† indicates below 75% of the recommended nutrient intake provided by total diet. The percent of daily calories from saturated fat is provided in place of the RDA/AI, as the *Dietary Guidelines for Americans, 2020–2025* recommends limiting saturated fat intake to <10% of daily calories.

**Table 6:** Nutrient implications of replacing Whole Grains with Starchy Vegetables in men aged 51 + years (2,000 kcal)^a^.

	Current pattern (reference)		Whole Grains: −2 oz-eq Starchy Vegetables: +1 cup-eq		Whole Grains: −10 oz-eq Starchy Vegetables: +5 cup-eq		Whole Grains: −21 oz-eq Starchy Vegetables: +10.5 cup-eq	
Refined Grains (oz-eq)	21		21		21		21	
Whole Grains (oz-eq)	21		19		11		0	
Starchy Vegetables (cup-eq)	5		6		10		15.5	
	Intake	% RDA/AI	Intake	% RDA/AI	Intake	% RDA/AI	Intake	% RDA/AI
Macronutrients								
Calories (kcal)	2001	100%	1998.9	100%	1990.6	100%	1979.2	99%
Protein (g)	92	164%	89.4	160%	78.9	141%	64.4	115%
Total fat (g)	71		70.6		69.2		67.2	
Carbohydrate (g)	259	199%	261.9	201%	273.7	211%	289.9	223%
Fiber (g)	30	107%	29.1	104%	25.3	**90%**	20.0	**72%†**
Cholesterol (mg)	214	**71%†**	213.1	**71%†**	209.6	**70%†**	204.8	**68%†**
Saturated fat (g)	18	–	17.8	–	17.0	–	15.8	–
% of daily calories		8.1%		8.0%		7.7%		7.2%
Monounsaturated fat (g)	25	–	25.8	–	29.0	–	33.3	–
Polyunsaturated fat (g)	22	–	21.2	–	17.8	–	13.1	–
Minerals								
Calcium (mg)	1,278	128%	1223.4	122%	1005.2	101%	705.0	**71%†**
Iron (mg)	14	175%	11.9	148%	3.3	**41%†**	−8.5	**0%†**
Magnesium (mg)	358	**85%**	337.4	**80%**	255.2	**61%†**	142.1	**34%†**
Phosphorus (mg)	1,654	236%	1582.1	226%	1294.3	185%	898.5	128%
Potassium (mg)	3,390	100%	3813.3	112%	5506.6	162%	7834.9	230%
Sodium (mg)	1,658	**72%†**	1589.5	**69%†**	1315.5	**57%†**	938.6	**41%†**
Zinc (mg)	13	118%	11.5	104%	5.5	**50%†**	−2.9	**0%†**
Copper (mg)	1.4	156%	1.4	158%	1.5	167%	1.6	179%
Selenium (mcg)	113	205%	100.7	183%	51.4	93%	−16.4	**0%†**
Vitamins								
Vitamin A (mcg RAE)	898	100%	844.8	94%	632.1	**70%†**	339.5	**38%†**
Vitamin E (mg AT)	10	**67%†**	9.7	**64%†**	8.3	**55%†**	6.4	**43%†**
Vitamin D (IU)	300	**50%†**	291.7	**49%†**	258.7	**43%†**	213.3	**36%†**
Vitamin C (mg)	129	143%	140.0	156%	183.8	204%	244.1	271%
Thiamin (mg)	1.8	150%	1.8	151%	1.9	154%	1.9	159%
Riboflavin (mg)	2	154%	2.0	152%	1.9	146%	1.8	138%
Niacin (mg)	23	144%	22.6	141%	20.9	130%	18.5	116%
Vitamin B6 (mg)	2.2	129%	2.4	139%	3.0	176%	3.9	228%
Vitamin B-12 (mcg)	6.2	258%	5.8	242%	4.2	175%	2.0	**83%**
Choline (mg)	355	**65%†**	364.2	**66%†**	400.9	**73%†**	451.4	**82%**
Vitamin K (mcg)	140	117%	143.7	120%	158.5	132%	178.9	149%
Folate (mcg DFE)	513	128%	452.5	113%	210.5	**53%†**	−122.4	**0%†**

a Bold indicates between 75% and 90% of the recommended nutrient intake provided by the total diet. Bold† indicates below 75% of the recommended nutrient intake provided by the total diet. The percent of daily calories from saturated fat is provided in place of the RDA/AI, as the *Dietary Guidelines for Americans, 2020–2025* recommends limiting saturated fat intake to <10% of daily calories.

### 3.4 Modeling objective 4: nutritional implications of modifications to both Refined Grains and Whole Grains and Starchy Vegetable quantities

When both Refined and Whole Grains were simultaneously replaced with Starchy Vegetables, the percent of nutrient recommendations met increased for choline and decreased for calcium, iron, sodium, zinc, selenium, riboflavin, folate, and vitamins A, B-12, D, and E in both women aged 19–30 years (Table 7) and men aged 51 + years (Table 8). Fiber, potassium, copper, and vitamins B6 and C also increased at all levels of replacement, but their intakes already met or exceeded the recommended amount in the reference pattern.

**Table 7:** Nutrient implications of simultaneous replacement of Refined and Whole Grains with Starchy Vegetables in women aged 19–30 years (2,000 kcal)^a^.

	Current pattern (reference)		Grains: −2 oz-eq Starchy Vegetables: +1 cup-eq		Grains: −10 oz-eq Starchy Vegetables: +5 cup-eq		Grains: −42 oz-eq Starchy Vegetables: +21 cup-eq	
Refined Grains (oz-eq)	21		21		21		21	
Whole Grains (oz-eq)	21		19		11		0	
Starchy Vegetables (cup-eq)	5		6		10		15.5	
	Baseline	% RDA/AI	Amount	% RDA/AI	Amount	% RDA/AI	Amount	% RDA/AI
Macronutrients								
Calories (kcal)	2001	100%	2003.7	100%	2014.7	101%	2058.5	103%
Protein (g)	92	200%	90.3	196%	83.4	181%	55.9	121%
Total fat (g)	71		71.0		70.8		70.0	
Carbohydrate (g)	259	199%	262.4	202%	276.2	212%	331.0	255%
Fiber (g)	30	107%	30.6	109%	33.1	118%	42.8	153%
Cholesterol (mg)	214	**71%†**	213.2	**71%†**	209.8	**70%†**	196.4	**65%†**
Saturated fat (g)	18	-	17.8	–	16.8	–	12.8	–
% of daily calories		8.1%		8.0%		7.5%		5.6%
Monounsaturated fat (g)	25	–	25.8	–	28.8	–	41.0	–
Polyunsaturated fat (g)	22	–	21.5	–	19.5	–	11.3	–
Minerals								
Calcium (mg)	1,278	128%	1234.9	123%	1062.7	106%	373.7	**37%†**
Iron (mg)	14	**78%**	12.5	**69%†**	6.4	**35%†**	−18.1	**0%†**
Magnesium (mg)	358	115%	358.7	116%	361.7	117%	373.3	120%
Phosphorus (mg)	1,654	236%	1626.9	232%	1518.4	217%	1084.3	155%
Potassium (mg)	3,390	130%	3871.7	149%	5798.3	223%	13504.9	519%
Sodium (mg)	1,658	**72%†**	1559.6	**68%†**	1166.0	**51%†**	−408.4	**0%†**
Zinc (mg)	13	163%	12.3	154%	9.5	119%	−1.7	**0%†**
Copper (mg)	1.4	156%	1.5	163%	1.8	194%	2.9	319%
Selenium (mcg)	113	205%	101.2	184%	54.0	98%	−134.8	**0%†**
Vitamins								
Vitamin A (mcg RAE)	898	128%	865.9	124%	737.4	105%	223.5	**32%†**
Vitamin E (mg AT)	10	**67%†**	9.9	**66%†**	9.4	**62%†**	7.3	**48%†**
Vitamin D (IU)	300	**50%†**	293.7	**49%†**	268.7	**45%†**	168.3	**28%†**
Vitamin C (mg)	129	172%	140.1	187%	184.4	246%	361.7	482%
Thiamin (mg)	1.8	164%	1.8	161%	1.7	150%	1.2	106%
Riboflavin (mg)	2	182%	1.9	176%	1.7	155%	0.7	**67%†**
Niacin (mg)	23	164%	22.9	164%	22.5	160%	20.7	148%
Vitamin B6 (mg)	2.2	169%	2.4	186%	3.3	254%	6.8	525%
Vitamin B-12 (mcg)	6.2	258%	6.0	248%	5.0	206%	1.0	**40%†**
Choline (mg)	355	**84%**	368.2	**87%**	421.2	99%	633.0	149%
Vitamin K (mcg)	140	156%	144.5	161%	162.6	181%	234.9	261%
Folate (mcg DFE)	513	128%	452.5	113%	210.3	**53%†**	−758.6	**0%†**

a Bold indicates between 75% and 90% of the recommended nutrient intake provided by the total diet. Bold† indicates below 75% of the recommended nutrient intake provided by the total diet. The percent of daily calories from saturated fat is provided in place of the RDA/AI, as the *Dietary Guidelines for Americans, 2020–2025* recommends limiting saturated fat intake to <10% of daily calories.

**Table 8:** Nutrient implications of simultaneous replacement of Refined and Whole Grains with Starchy Vegetables in men aged 51 + years (2,000 kcal)^a^.

	Current pattern (reference)		Grains: −2 oz-eq Starchy Vegetables: +1 cup-eq		Grains: −10 oz-eq Starchy Vegetables: +5 cup-eq		Grains: −42 oz-eq Starchy Vegetables: +21 cup-eq	
Refined Grains (oz-eq)	21		21		21		21	
Whole Grains (oz-eq)	21		19		11		0	
Starchy Vegetables (cup-eq)	5		6		10		15.5	
	Baseline	% RDA/AI	Baseline	% RDA/AI	Baseline	% RDA/AI	Baseline	% RDA/AI
Macronutrients								
Calories (kcal)	2001	100%	2003.7	100%	2014.7	101%	2058.5	103%
Protein (g)	92	164%	90.3	161%	83.4	149%	55.9	100%
Total fat (g)	71		71.0		70.8		70.0	
Carbohydrate (g)	259	199%	262.4	202%	276.2	212%	331.0	255%
Fiber (g)	30	107%	30.6	109%	33.1	118%	42.8	153%
Cholesterol (mg)	214	**71%†**	213.2	**71%†**	209.8	**70%†**	196.4	**65%†**
Saturated fat (g)	18	–	17.8	–	16.8	–	12.8	–
% of daily calories		8.1%		8.0%		7.5%		5.6%
Monounsaturated fat (g)	25		25.8		28.8		41.0	
Polyunsaturated fat (g)	22		21.5		19.5		11.3	
Minerals								
Calcium (mg)	1,278	128%	1234.9	123%	1062.7	106%	373.7	**37%†**
Iron (mg)	14	175%	12.5	156%	6.4	*79%*	−18.1	**0%†**
Magnesium (mg)	358	**85%**	358.7	**85%**	361.7	**86%**	373.3	**89%**
Phosphorus (mg)	1,654	236%	1626.9	232%	1518.4	217%	1084.3	155%
Potassium (mg)	3,390	100%	3871.7	114%	5798.3	171%	13504.9	397%
Sodium (mg)	1,658	**72%†**	1559.6	**68%†**	1166.0	**51%†**	−408.4	**0%†**
Zinc (mg)	13	118%	12.3	112%	9.5	**86%**	−1.7	**0%†**
Copper (mg)	1.4	156%	1.5	163%	1.8	194%	2.9	319%
Selenium (mcg)	113	205%	101.2	184%	54.0	98%	−134.8	**0%†**
Vitamins								
Vitamin A (mcg RAE)	898	100%	865.9	96%	737.4	**82%**	223.5	**25%†**
Vitamin E (mg AT)	10	**67%†**	9.9	**66%†**	9.4	**62%†**	7.3	**48%†**
Vitamin D (IU)	300	**50%†**	293.7	**49%†**	268.7	**45%†**	168.3	**28%†**
Vitamin C (mg)	129	143%	140.1	156%	184.4	205%	361.7	402%
Thiamin (mg)	1.8	150%	1.8	148%	1.7	138%	1.2	98%
Riboflavin (mg)	2	154%	1.9	149%	1.7	131%	0.7	**57%†**
Niacin (mg)	23	144%	22.9	143%	22.5	140%	20.7	129%
Vitamin B6 (mg)	2.2	129%	2.4	142%	3.3	194%	6.8	401%
Vitamin B-12 (mcg)	6.2	258%	6.0	248%	5.0	206%	1.0	**40%†**
Choline (mg)	355	**65%†**	368.2	**67%†**	421.2	**77%**	633.0	115%
Vitamin K (mcg)	140	117%	144.5	120%	162.6	136%	234.9	196%
Folate (mcg DFE)	513	128%	452.5	113%	210.3	**53%†**	−758.6	**0%†**

a Bold indicates between 75% and 90% of the recommended nutrient intake provided by the total diet. Bold† indicates below 75% of the recommended nutrient intake provided by the total diet. The percent of daily calories from saturated fat is provided in place of the RDA/AI, as the *Dietary Guidelines for Americans, 2020–2025* recommends limiting saturated fat intake to <10% of daily calories.

## 4 Discussion

The results of this modeling analysis demonstrate that the recommended daily serving of White Potatoes provides 11% of daily potassium and 6% of daily fiber – both of which are nutrients of public health concern – as well as vitamins B6 and C, copper, magnesium, thiamin, and niacin. The differences in the nutrient contribution of Whole and Refined Grains compared to White Potatoes and Starchy Vegetables demonstrate that Starchy Vegetables and Grains are not interchangeable. Replacing Refined Grains with Starchy Vegetables increases fiber, potassium, vitamin C, and choline intake but decreases other nutrients such as iron, folate, and riboflavin – all of which are enriched or fortified in Refined Grains. While replacing Whole Grains with Starchy Vegetables similarly increases potassium, vitamin C, and choline, this replacement decreases the intake of fiber, as well as calcium, iron, magnesium, folate, and other vitamins. Previous modeling analyses have similarly found that Grains and Starchy Vegetables are not interchangeable. For instance, replacement of Starchy Vegetables with Grains in a sample menu consistent with the DGA resulted in decreases of 10% or more in potassium, fiber, and vitamins B6 and C (17). Conversely, studies modeling the removal of grains have found decreases in other key micronutrients, particularly those such as folic acid, iron, thiamin, niacin, and riboflavin provided by enriched and fortified products (18, 19). It is also important to note that Starchy Vegetables and Grains provide different types of fiber (e.g., pectin, lignin, beta-glucans, and hemicelluloses such as arabinoxylan), which are metabolized differently in the gastrointestinal tract and have different phenolic content (20, 21). Thus, both Starchy Vegetables and Grain foods are important contributors to the diet.

Notably, the 2025 DGAC concluded that their FPM analyses supported “exploring a flexibility that increases Starchy Vegetables (including Starchy Red and Orange Vegetables) above the proposed quantities in a healthy dietary pattern while simultaneously decreasing Total Grains” (14). However, the final Eat Healthy Your Way dietary pattern proposed by the 2025 DGAC decreased Starchy Vegetables with no change to Total Grains (12). Since potatoes are a good source of potassium, reducing the recommended servings of Starchy Vegetables is inconsistent with potassium’s classification as a nutrient of public health concern. According to the 2025 DGAC, over 70% of Americans consume potassium below the recommended level (12). Boiled and baked potatoes (two of the most commonly consumed forms of potatoes) provide up to 1.5× more potassium than other top-consumed Starchy Vegetables (corn and peas) per MyPlate cup eq. (1, 12). Only four vegetables have higher potassium content in a standard portion than the 926 mg provided by one medium potato (beet greens: 1309 mg; fufu: 1080 mg; swiss chard: 961 mg; and lima beans: 955 mg) (22); however, it is important to note that these vegetables are not frequently consumed in the U.S. Although Starchy Vegetables are underconsumed by 86% of Americans, this analysis indicates that potatoes account for 6–8% of actual potassium intake. Similarly, NHANES analyses have found that potatoes contribute 4.0–6.7% of daily potassium in children and adolescents (23–25) and 6.7% in adults (26). Notably, the 2025 DGAC called out Starchy Vegetables (of which potatoes comprise the majority of consumption) as a top food category source of potassium intake among Americans aged 2 years and older (12). With most Americans underconsuming vegetables and not meeting potassium recommendations, potato intake plays a critical role in food group and nutrient contributions.

In addition to their nutrient density, potatoes are a preferred vegetable among Americans and have the potential to improve diet quality. The FPM analyses by the 2020 and 2025 DGACs demonstrate that Starchy Vegetables account for over 25% of vegetable intake among Americans aged 2 years and older. Within the Starchy Vegetable subgroup, 80–86% of intake comes from various forms of potatoes. Cluster analyses have demonstrated that food patterns with varying levels of potato intake (8.6–12% of daily calorie intake) can be associated with both higher and lower diet quality in children and adults, with differences likely due to the presence or absence of other food categories in the dietary pattern given the finite range of calories from potatoes across the clusters (7). Additional NHANES assessments have demonstrated that adolescent potato consumers (including baked or boiled potatoes, mashed potatoes, and potato mixtures, fried potatoes, and potato chips/crisps) have higher Healthy Eating Index (HEI-2015) total scores, as well as subcomponent scores for total vegetables (6). A cafeteria-based study also found that children consumed more peas and carrots when they were combined with smiley-face-shaped potatoes (27). In clinical intervention studies among adults, incorporating potatoes into the diet had no detrimental effects on cardiometabolic risk factors compared to almonds (11) or refined grains (10) and improved diet quality compared to an isocaloric amount of refined grains (10).

Cost, convenience, and culture are additional important considerations for the implementation of dietary guidelines. The 2021 Thrifty Food Plan lists potatoes, corn, lima beans, plantain, and cassava as lower-cost Starchy Vegetables (28). Among these, potatoes are by far the most commonly consumed. According to the 2025 DGAC FPM Report (4), white and yellow corn comprise only 8.1% of the Starchy Vegetable subgroup consumption, and plantains (1.2%), lima beans (0.6%), and cassava (0.7%) are consumed relatively rarely compared to baked, boiled, and mashed white potatoes. Additionally, research using the Nutrient Rich Foods Index—a scoring system that ranks foods based on their nutrient content—and USDA food price datasets identified potatoes as one of the foods with the most favorable nutrient-to-price ratio based on nutrients to encourage (potassium, fiber, protein, vitamins A, C and E, calcium, iron and magnesium) and nutrients to limit (saturated fat, added sugar, and sodium) (29). Among vegetables included in the National School Lunch Program, white potatoes and beans provided the best nutritional value. White potatoes were among the top two lowest-cost sources of potassium, fiber, and vitamin C, with median costs per 10% daily value being $0.14, $0.19, and $0.10, respectively (30). Not only are potatoes affordable, they are also foundational to many foodways enjoyed throughout the United States and commonly found in Asian, African, Latin American, and European heritage diets.

Effective implementation of dietary guidance to help improve diet quality and close pervasive gaps in vegetable consumption and associated nutrients remains a challenge. Previous iterations of the DGA have reinforced that dietary guidance can be most effective when it focuses on small shifts in current behaviors rather than recommendations that require drastic changes to dietary habits (2). As such, the concept of “meeting people where they are” was a common theme throughout the 2025 DGAC process. Flexibilities, such as those recommended by the 2025 DGAC FPM for Starchy Vegetables and Grains, allow for dietary patterns to be adapted according to personal preferences, cultural norms, budget, and/or dietary restrictions or sensitivities. Potatoes are a nutrient-dense vegetable that is affordable, convenient, and “flexible,” supporting a healthy dietary pattern across multiple cultural foodways in a variety of preparations.

The major strengths of this study include mirroring the FPM approach used by the 2025 DGAC. This analysis also calculated the recommended and actual intake of White Potatoes within the Starchy Vegetables subgroup, as potatoes are the most commonly consumed type of Starchy Vegetable. The serving recommendation for White Potatoes was also reported as “per day,” which provides more real-world application for consumers than per-week servings. As in any dietary modeling analysis, the results represent an academic exercise that evaluates the maximum effect of modeling and may not reflect actual individual dietary behavior. This type of analysis also does not consider the feasibility of modelled dietary changes. Some of the modeling scenarios replacing Refined and/or Whole Grains also call for more drastic changes that may not be achievable for consumers in a real-world scenario.

### 4.1 Conclusion

The results of this modeling study provide insight into the unique nutritional contribution of White Potatoes. One MyPlate equivalent of White Potatoes provides more potassium, copper, choline, and vitamins B6, C, and K than one MyPlate equivalent of Grains. Comparatively, one MyPlate equivalent of Grains provides more calcium, iron, zinc, selenium, riboflavin, folate, and vitamins A, B-12, and D. Replacement of Refined or Whole Grains with Starchy Vegetables increases nutrients such as potassium and vitamin C but decreases other nutrients such as iron, folate, riboflavin, and other vitamins. Therefore, due to their distinct nutritional profiles, Starchy Vegetables and Grains are not interchangeable in dietary patterns, as both provide important nutrients. These results suggest that reducing the recommended intake of Starchy Vegetables may decrease the intake of key nutrients such as potassium and be counterproductive for improving diet quality and achieving healthier dietary patterns among Americans. Observational and clinical studies are needed to confirm the results of this modeling analysis.

## Data Availability

The raw data supporting the conclusions of this article will be made available by the authors, without undue reservation.

## References

[1] United States Department of Agriculture. FoodData Central. Available online at: https://fdc.nal.usda.gov/

[2] U.S. Department of Agriculture and U.S. Department of Health and Human Services Dietary guidelines for Americans, 2020–2025 (2020)

[3] Dietary Guidelines Advisory Committee and Food Pattern Modeling Team. 2020 dietary guidelines advisory committee food pattern modeling report: Ages 2 years and older. Washington, D.C.: U.S. Department of Agriculture (2020).

[4] Taylor CA Eicher-Miller HA Abrams SA Booth SL Byrd-Bredbenner C Fung T . Should foods and beverages with lower nutrient density (i.e., those with added sugars, saturated fat, and sodium) contribute to item clusters, representative foods, and therefore the nutrient profiles for each food group and subgroup used in modeling the USDA dietary patterns? Food pattern modeling report. US Department of Agriculture, Food and Nutrition Service, Center for Nutrition Policy and Promotion, Nutrition and Economic Analysis Branch. (2024)

[5] U.S. Department of Agriculture, Economic Research Service. U.S. per capita loss-adjusted vegetable availability, (2019). Available online at: https://www.ers.usda.gov/data-products/ag-and-food-statistics-charting-the-essentials/food-availability-and-consumption

[6] Agarwal S Fulgoni VL. Intake of potatoes is associated with higher diet quality, and improved nutrient intake and adequacy among US adolescents: NHANES 2001-2018 analysis. Nutrients. (2021) 13:2614. doi: 10.3390/nu13082614, PMID: 34444775 PMC8400280

[7] Fulgoni K Fulgoni VL. Certain dietary patterns including potatoes are associated with higher and lower diet quality and physiological measures in children and adults, NHANES 2001-2018. Front Nutr. (2022) 9:987861. doi: 10.3389/fnut.2022.987861, PMID: 36438770 PMC9684662

[8] Djousse L Zhou X Lim J Kim E Sesso HD Lee IM . Potato consumption and risk of type 2 diabetes mellitus: a harmonized analysis of 7 prospective cohorts. J Nutr. (2024) 154:3079–87. doi: 10.1016/j.tjnut.2024.07.020, PMID: 39289134 PMC12612587

[9] Djousse L Zhou X Lim J Kim E Sesso HD Lee IM . Potato consumption and risk of cardiovascular disease in a harmonized analysis of seven prospective cohorts. Nutrients. (2025) 17:451. doi: 10.3390/nu17030451, PMID: 39940309 PMC11820226

[10] Johnston EA Petersen KS Kris-Etherton PM. Daily intake of non-fried potato does not affect markers of glycaemia and is associated with better diet quality compared with refined grains: a randomised, crossover study in healthy adults. Br J Nutr. (2020) 123:1032–42. doi: 10.1017/S0007114520000252, PMID: 31964428 PMC7282869

[11] Smith DL Hanson RL Dickinson SL Chen X Goss AM Cleek JB . French-fried potato consumption and energy balance: a randomized controlled trial. Am J Clin Nutr. (2022) 115:1626–36. doi: 10.1093/ajcn/nqac045, PMID: 35179193 PMC9170465

[12] Dietary Guidelines Advisory Committee. Scientific report of the 2025 dietary guidelines advisory committee: advisory report to the secretary of health and human services and secretary of agriculture. HHS and USDA; (2024). Available online at: https://www.dietaryguidelines.gov/2025-advisory-committee-report

[13] Dietary Guidelines Advisory Committee. (2025). Proposed scientific questions. Available online at: https://www.dietaryguidelines.gov/sites/default/files/2022-07/Proposed%20Scientific%20Questions_508c_Final.pdf

[14] Taylor CA Fung T Booth SL Abrams SA Byrd-Bredbenner C . (2024). What are the implications for nutrient intakes when modifying the quantities of the grains group within the healthy U.S.-style dietary pattern? What are the implications for nutrient intakes when specific individual staple grains are emphasized; or when grains are replaced with other staple carbohydrate foods (i.e., starchy vegetables; beans, peas, and lentils; starchy red and Orange vegetables)? Food pattern modeling report. U.S. Department of Agriculture, Food and Nutrition Service, Center for Nutrition Policy and Promotion. Nutrition and economic analysis branch

[15] U.S. Department of Agriculture, Agricultural Research Service. Food products equivalents database. Available online at: https://www.ars.usda.gov/northeast-area/beltsville-md-bhnrc/beltsville-human-nutrition-research-center/food-surveys-research-group/docs/fped-databases/

[16] Dietary Guidelines Advisory Committee and Data Analysis Team. Data supplement for food group and nutrient distribution: All life stages. Washington, DC: U.S. Department of Agriculture and U.S. Department of Health and Human Services (2020).

[17] Ayoob KT. Carbohydrate confusion and dietary patterns: unintended public health consequences of food swapping. Front Nutr. (2023) 10:1266308 37841395 10.3389/fnut.2023.1266308 PMC10568005

[18] Estell ML Barrett EM Kissock KR Grafenauer SJ Jones JM Beck EJ. Fortification of grain foods and NOVA: the potential for altered nutrient intakes while avoiding ultra-processed foods. Eur J Nutr. (2022) 61:935–45. doi: 10.1007/s00394-021-02701-1, PMID: 34668030

[19] Berner LA Keast DR Bailey RL Dwyer JT. Fortified foods are major contributors to nutrient intakes in diets of US children and adolescents. J Acad Nutr Diet. (2014) 114:1009–1022.e8. doi: 10.1016/j.jand.2013.10.012, PMID: 24462266

[20] Carlsen H Pajari AM. Dietary fiber – a scoping review for Nordic nutrition recommendations 2023. Food Nutr Res (2023); 67. Available online at: https://foodandnutritionresearch.net/index.php/fnr/article/view/9979 10.29219/fnr.v67.9979 PMC10619389 37920675

[21] Popoola-Akinola OO Raji TJ Olawoye B. Lignocellulose, dietary fibre, inulin and their potential application in food. Heliyon. (2022) 8:e10459. doi: 10.1016/j.heliyon.2022.e10459, PMID: 36090233 PMC9449745

[22] Dietary Guidelines for Americans. Food Sources of Potassium: Nutrient-dense Food and Beverage Sources, Amounts of Potassium and Energy per Standard Portion. Available online at: https://www.dietaryguidelines.gov/food-sources-potassium

[23] Leme AC Baranowski T Thompson D Philippi S CE ON Fulgoni VL . Food sources of shortfall nutrients among US adolescents: National Health and nutrition examination survey (NHANES) 2011-2014. Fam Community Health. (2020) 43:59–73. doi: 10.1097/FCH.0000000000000243 31764307

[24] O’Neil CE Nicklas TA Fulgoni VL. Food sources of energy and nutrients of public health concern and nutrients to limit with a focus on milk and other dairy foods in children 2 to 18 years of age: national health and nutrition examination survey, 2011−2014. Nutrients. (2018) 10:1050. 30096892 10.3390/nu10081050 PMC6116120

[25] Keast DR Fulgoni VL Nicklas TA O’Neil CE. Food sources of energy and nutrients among children in the United States: National Health and nutrition examination survey 2003–2006. Nutrients. (2013) 5:283–301. doi: 10.3390/nu5010283, PMID: 23340318 PMC3571649

[26] O’Neil CE Keast DR Fulgoni VL Nicklas TA. Food sources of energy and nutrients among adults in the US: NHANES 2003–2006. Nutrients. (2012) 4:2097–120. 23363999 10.3390/nu4122097 PMC3546624

[27] Hernandez Sanchez MG Bellini S Christensen WF Jefferies LK LeCheminant JD Patten EV . The effects of potato presentation on vegetable intake in school-aged children: a cross-over study. Nutrients. (2023) 15:4496. doi: 10.3390/nu15214496, PMID: 37960149 PMC10650674

[28] U.S. Department of Agriculture. Thrifty food plan, 2021. (2021) Report No.: FNS-916.

[29] Drewnowski A. The nutrient rich foods index helps to identify healthy, affordable foods. Am J Clin Nutr. (2010) 91:1095S–101S. doi: 10.3945/ajcn.2010.28450D, PMID: 20181811

[30] Drewnowski A Rehm CD. Vegetable cost metrics show that potatoes and beans provide most nutrients per penny. PLoS One. (2013) 8:e63277. doi: 10.1371/journal.pone.0063277, PMID: 23691007 PMC3654977

