# Supplementary material for: Assessment of the unique nutrient contribution of white potatoes in the diet and the nutrient implications of replacing Refined and Whole Grains with Starchy Vegetables
Source: Front Nutr. 2025 Nov 27;12:1692564. doi: 10.3389/fnut.2025.1692564 (PMC12695571; doi:10.3389/fnut.2025.1692564)
Supplement: Supplementary file 1 [file Table_1.DOCX]

Supplementary Material

# Supplementary Tables

**Supplementary Table 1.** **Comparison of nutrient content of grains with starchy vegetable subgroups^1^**

| **Comparison Scenario** | 2 oz-eq Grains vs  1 cup-eq Starchy Vegetables | 2 oz-eq Grains vs  1 cup-eq White Potatoes |
| --- | --- | --- |
|  | **Amount Difference** | **Amount Difference** |
| **Macronutrients** |  |  |
| Calories (kcal) | 2.74 | 7.8 |
| Protein (g) | -1.72 | -2.1 |
| Total fat (g) | -0.05 | 0.22 |
| Carbohydrate (g) | 3.43 | 3.89 |
| Fiber (g) | 0.61 | 0.32 |
| Cholesterol (mg) | -0.84 | -0.84 |
| Saturated fat (g) | -0.25 | -0.21 |
| Monounsaturated fat (g) | 0.76 | 1.02 |
| Polyunsaturated fat (g) | -0.51 | -0.52 |
| **Minerals** |  |  |
| Calcium (mg) | -43.06 | -43.67 |
| Iron (mg) | -1.53 | -1.57 |
| Magnesium (mg) | 0.73 | -1.77 |
| Phosphorus (mg) | -27.13 | -34.26 |
| Potassium (mg) | 481.66 | 520.1 |
| Sodium (mg) | -98.4 | -82.65 |
| Zinc (mg) | -0.7 | -0.8 |
| Copper (mg) | 0.07 | 0.08 |
| Selenium (mcg) | -11.8 | -11.75 |
| **Vitamins** |  |  |
| Vitamin A (mcg RAE) | -32.12 | -37.34 |
| Vitamin E (mg AT) | -0.13 | -0.09 |
| Vitamin D (IU) | -6.27 | -6.27 |
| Vitamin C (mg) | 11.08 | 10.87 |
| Thiamin (mg) | -0.03 | -0.02 |
| Riboflavin (mg) | -0.06 | -0.08 |
| Niacin (mg) | -0.11 | -0.08 |
| Vitamin B6 (mg) | 0.22 | 0.24 |
| Vitamin B-12 (mcg) | -0.25 | -0.25 |
| Choline (mg) | 13.24 | 9.22 |
| Vitamin K (mcg) | 4.52 | 3.49 |
| Folate (mcg DFE) | -60.55 | -66.04 |

^1^ Total Grains nutrient values were calculated as the sum of 1 oz-eq of Whole Grains and 1 oz-eq of Refined Grains. Italics indicates a ≥50% decrease in nutrient content compared to 2 oz-eq of Grains. Bolding indicates ≥50% increase in nutrient content compared to 2 oz-eq of Grains.
